# The relationship between openness to experience and humor production: Exploring the mediating roles of cognitive flexibility and ambiguity tolerance

**DOI:** 10.1002/pchj.799

**Published:** 2024-09-16

**Authors:** Cuicui Sun, Jielin Shen, Jiajia Lin, Tingyu Zhang, Junyi Li

**Affiliations:** ^1^ School of Psychology Sichuan Normal University Chengdu China

**Keywords:** ambiguity tolerance, cognitive flexibility, humor production, openness to experience, sense of humor

## Abstract

The purpose of this study was to examine how individual openness to experience influences humor production and to explore the underlying psychological mechanisms of this relationship, specifically focusing on cognitive flexibility (the cognitive path) and ambiguity tolerance (the motivational path). To comprehensively evaluate individuals' humor production ability, Study 1 employed a subjective self‐report questionnaire on sense of humor, while Study 2 used an objective humor dialogue generation task. The results of Study 1 indicated that openness to experience did not directly impact sense of humor; instead, the relationship between openness to experience and sense of humor was fully mediated by cognitive flexibility. In Study 2, findings showed that openness to experience positively predicted humor production ability, with ambiguity tolerance partially mediating this effect. These results suggest that individuals with higher levels of openness to experience have a greater capacity for generating humorous perspectives. Moreover, the study identified two psychological pathways—cognition and motivation—in the process of generating funny ideas. The specific pathway influenced by the measurement method used for humor production further highlights the importance of both cognitive flexibility and ambiguity tolerance in understanding how openness to experience contributes to humor production.

## INTRODUCTION

Humor is generally perceived to be indicative of good courtship, intelligence, and mental health (Kaufman et al., 2008) and is comprised of both passive humor perception (humor appreciation and comprehension) and active humor production. Humor perception is related to the recipient and involves both a cognitive and an emotional dimension, focusing on whether the receiver “gets the joke” (the cognitive dimension) and “enjoys the joke” (the emotional dimension) (Gardner et al., 1975). Humor production is pertinent to the creator and primarily involves “the ability or habit of individuals to actively generate new humor examples or make others laugh, as well as the internal process of humor generation” (Sun et al., 2023). Considered a coveted social ability, humor production plays a central role in our everyday lives. However, compared to humor appreciation or comprehension, research on humor production is relatively scarce (Ruch & Heintz, 2019).

Existing research on humor production primarily focuses on the relationship between individual characteristics and humor production (Greengross et al., 2012; Kaufman et al., 2008; Kozbelt & Nishioka, 2010). Notably, personality traits, such as the Big Five personality factors (extraversion, neuroticism, agreeableness, conscientiousness, and openness to experience) have been widely discussed as predictive variables. Among the Big Five, only openness to experience has a clearly confirmed positive impact on humor production. The influence of other traits, such as extraversion, on humor production is either insignificant or remains controversial (Greengross et al., 2012; Köhler & Ruch, 1996; Nusbaum et al., 2017; Sutu et al., 2021). Researchers believe that individuals with high openness to experience typically exhibited higher verbal fluency and an ability to appreciate unconventional aspects of humor, which facilitates the generation and creation of humorous content.

Despite the well‐established positive effect of openness to experience on humor production (Greengross et al., 2012; Sutu et al., 2021), the underlying reasons for the heightened humor generation abilities observed in individuals with high openness to experience remain theoretical, lacking empirical evidence at present. Therefore, this study aims to address this gap by building upon previous research to uncover the psychological mechanisms through which openness to experience enhances humor production. Specifically, it focuses on exploring the roles of cognitive flexibility (the cognitive path) and tolerance for ambiguity (the motivational path).

## LITERATURE REVIEW

Openness to experience is typically regarded as one of the internal psychological dimensions of an individual, describing the differences in individual thinking structure and function (McCrae, 1993). It is reflected in the breadth, depth, and permeability of one's consciousness, as well as the need to constantly expand and explore experience (Fürst et al., 2016). High levels of openness to experience are linked to greater knowledge, higher association ability, and language fluency. This is usually beneficial for humor production, which tends to involve the use of original ideas, verbal ability, and unconventional thinking. A meta‐analysis of the correlation between personality traits and humor production revealed that only openness to experience had a significant association with humor production (*r* = .23) (Nusbaum, 2015). Subsequent studies carried out with different Big Five personality scales and various humor production tasks revealed that, after controlling for the other Big Five personality traits, openness of individuals to experience could still significantly predict humor production ability, with predictive effect values reaching a medium‐high level (*β* = 0.36–0.54) (Nusbaum et al., 2017). Other researchers found similar results (Greengross et al., 2012; Sutu et al., 2021). For instance, Sutu et al. (2021) observed that openness to experience had the strongest predictive effect on humor production when compared to other individual predictors (socioeconomic status, intelligence, etc.).

Individuals who exhibit high levels of openness to experience are often associated with greater cognitive needs, integration complexity, and cognitive flexibility (McCrae, 1993). Cognitive flexibility refers to the ability to adapt cognitive processing strategies by generating new rules to cope with unpredictable environments. It involves transitioning between different perspectives, categories, and modes of thinking, and is closely linked to attention processes (Moore & Malinowski, 2009). Cognitive flexibility is regarded as a crucial element for creativity as it enables individuals to challenge traditions, solve problems from fresh angles, and generate novel ideas. Humor production, being a unique form of verbal creativity, requires individuals to shift between various cognitive schemata and integrate seemingly unrelated or contradictory schemata (Ruch & Heintz, 2019). Therefore, we propose that cognitive flexibility may serve as the psychological mechanism through which individual openness to experience influences humor production ability.

In addition to cognitive abilities, highly open individuals also excel in humor production when it comes to their emotions, motivations, and attitudes. Research suggests that individuals with high levels of openness experience a broader range of emotions and possess nonconventional values (Silvia et al., 2015). Consequently, they are more adept at adopting inclusive and distinct perspectives, exhibiting greater tolerance toward future uncertainties. Ambiguity tolerance is a continuum ranging from positive to negative reactions, with ambiguity intolerance (AI) at one end and ambiguity tolerance at the other (McLain, 2009). AI refers to individuals perceiving ambiguous situations as threats or sources of discomfort. Conversely, tolerance for ambiguity refers to an individual's preference for and comfort with uncertain situations (Litman, 2005). Individuals with high ambiguity tolerance may be attracted to the mystery or cognitive challenge posed by ambiguous information. They interpret ambiguous situations more adequately and realistically, without denying or distorting their complexity. This motivation to tolerate ambiguity allows them to endure the discomfort of ambiguous situations long enough to adapt and generate more flexible responses.

Currently, there has been no direct investigation into the relationship between ambiguity tolerance and humor production ability. However, certain studies have shown that ambiguity tolerance has a positive impact on creative thinking, particularly in terms of divergent thinking (Stoycheva, 2010). Both humor production and creativity involve dealing with ill‐defined problems that are not easily resolved. To generate humorous ideas, individuals need to redefine or frame the problem in a specific way (Kozbelt & Nishioka, 2010). Humor, unlike other forms of problem‐solving, has loose requirements for feasibility and appropriateness of the response. As long as the response is amusing to the recipient, it can be considered interesting, even if it is odd or nonsensical. In other words, the potential range of individual responses in humor production is vast. The inherent ambiguity in the context of humor aligns with the nature of tasks where the answers are not unique. Therefore, we propose that ambiguity tolerance may serve as another crucial psychological mechanism through which individual openness to experience affects humor production ability.

**FIGURE 1:**
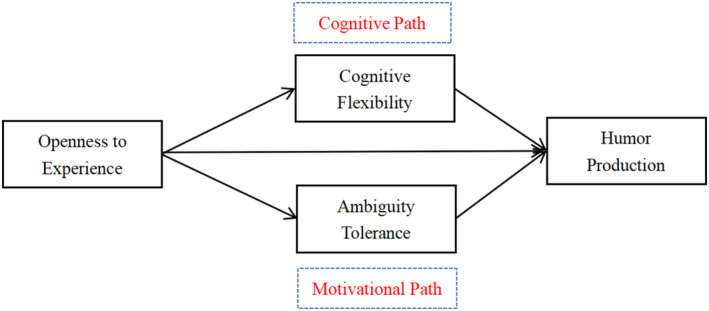
The hypothesis model.

In summary, individual openness to experience is positively associated with the ability to generate humor. Cognitive flexibility and tolerance for ambiguity may act as internal psychological mechanisms that mediate the relationship between openness to experience and humor production. Thus, this study presents a theoretical hypothesis model, as depicted in Figure 1.

## STUDY 1

### Methods

#### Participants

A total of 221 online questionnaires were distributed randomly to college students enrolled in two universities located in Chengdu and Wuhan of China. Prior to filling out the questionnaire, we obtained electronic informed consent from all participants. All procedures carried out in this and the following study were approved by the Ethics Committee of Sichuan Normal University. After removing questionnaires with incomplete or inconsistent responses, a total of 216 valid questionnaires were collected, resulting in a response rate of 98%. Among the participants, 47 (21.76%) were male, and 169 (78.24%) were female, with an average age of 19.88 ± 1.12 years. Upon completion of the questionnaire, participants received a cash reward as a token of appreciation.

To ensure the adequacy of the sample size, a power analysis was conducted using G*Power 3.1 software. The significance level (alpha) was set at 0.05, and the effect size was determined to be 0.15 based on previous research by Cohen (1988). A power of 0.95 was selected, and the number of predictors was set to 3. The analysis results indicated that a minimum sample size of 119 was required. Thus, our sample size of 216 was deemed satisfactory.

#### Measures

##### The openness scale

The openness scale used in this study is a Chinese adaptation (Wang & Cui, 2006) based on the Chinese localized personality scales (QZPS; Wang & Cui, 2003) and NEO‐PI‐R (McCrae & Costa Jr., 1985). The adapted scale consists of 36 items, which cover six dimensions: tolerance and experimentation, emotion and aesthetics, enjoyment of fantasy, rationality and speculation, wisdom and breadth, and novelty and reflection. Participants responded using a Likert scale with 5 points (1 = *disagree at all*, 5 = *completely agree*), with 16 items reverse‐scored. Higher scores indicate higher levels of openness to experience. The alpha coefficient of the scale in this study was .75.

##### Cognitive flexibility scale

In this study, we utilized the Cognitive Flexibility Scale created by Martin and Rubin (1995), which is composed of 12 items. The scale was rated on a 6‐point Likert scale (ranging from 1 = *disagree at all* to 6 = *completely agree*), with four items being reverse‐scored to ensure that higher scores reflected greater levels of cognitive flexibility. The alpha coefficient for the scale in our study was .79.

##### Ambiguity tolerance scale

We employed a scale developed by McLain (2009) that is based on the concept of ambiguity tolerance, which refers to the tendency to respond to complex, unfamiliar, and incomprehensible stimuli on a continuum ranging from aversion to attraction. The scale comprises 13 items and was rated using a 7‐point Likert scale (ranging from 1 = *completely inconsistent* to 7 = *completely consistent*), with nine items being reverse‐scored. Higher scores indicate a higher level of ambiguity tolerance. In our study, the scale showed good internal consistency with an alpha coefficient of .77.

##### Sense of humor questionnaire

The humor questionnaire utilized in this study was developed by Chen et al. (2011) specifically for Chinese undergraduates. The questionnaire consists of 23 items and covers five distinct dimensions of humor: humor entertainment (e.g., I often speak humorously and make people laugh), humor communication (e.g., when expressing dissatisfaction, I usually do so in a playful manner), humor coping (e.g., I often view difficulties and setbacks from a fun and relaxed perspective), humor appreciation (e.g., I frequently read humorous and interesting books), and humor learning (e.g., I observe the words and actions of humorous and witty people in my daily life). Participants rated each item on a 5‐point Likert scale, ranging from 1 (*completely inconsistent*) to 5 (*completely consistent*), with higher scores indicating a stronger sense of humor. The scale demonstrated high internal consistency in this study, with an alpha coefficient of .88.

#### Data analysis

Descriptive statistics and correlation analysis were conducted using SPSS 26.0. The mediating effect was tested using PROCESS 3.4.1 MODEL 4, and the indirect effect interval was examined using the Bootstrap program. The sample size in the model was set at 5000, and the confidence interval (CI) was set at 95%. If the CI does not include zero, the effect is considered significant; otherwise, it is not significant. Additionally, considering the gender imbalance in the selected sample in this study, we controlled for gender as a covariate in the statistical analysis.

### Results

#### Common method bias test

All data collected in this study came from students' subjective reports, which may have resulted in common method bias. Therefore, before conducting the formal data analysis, we first used Harman's single‐factor test to examine the common method bias (Zhou & Long, 2004). The results showed that the variance explained by the first factor was 13.11% (less than 40%), indicating that the common method bias was within an acceptable range.

#### Descriptive statistical analysis

Descriptive statistical analysis reveals the means, standard deviations, and inter‐variable correlations, as shown in Table 1. Notably, individual openness to experience is significantly and positively associated with ambiguity tolerance, cognitive flexibility, and sense of humor. Additionally, cognitive flexibility is significantly and positively correlated with ambiguity tolerance and sense of humor.

**TABLE 1:** Descriptive statistics and correlation analysis between variables (*N* = 216).

	*M* ± *SD*	1	2	3	4
1 Openness to experience	3.48 ± .32	1			
2 Ambiguity tolerance	3.53 ± .74	0.45**	1		
3 Cognitive flexibility	4.10 ± .62	0.49**	0.38**	1	
4 Sense of humor	3.68 ± .56	0.17*	0.05	0.38**	1

*Note*: **p < .01, *p < .05.

#### The mediating effect of cognitive flexibility and ambiguity tolerance

The parallel mediating effect of cognitive flexibility and ambiguity tolerance on the relationship between openness to experience and sense of humor was analyzed using Bootstrap method with 5000 resamples in PROCESS (Model 4) (Hayes, 2015). The mediating effect of cognitive flexibility on the relationship between openness to experience and sense of humor was significant with *β* = .20, and the 95% CI [.10, .31] did not include “0,” while the direct effect of openness to experience on sense of humor was not significant with *β* = .04, and the 95% CI [−.13, .21] included “0,” indicating that cognitive flexibility fully mediated the relationship between openness to experience and sense of humor. The mediating effect of ambiguity tolerance on the relationship between openness to experience and sense of humor was not significant with *β* = .04, and the 95% CI [−.14, .02] included “0,” indicating that ambiguity tolerance did not mediate the relationship between openness to experience and sense of humor. The relevant results are presented in Table 2.

**TABLE 2:** Mediation analysis results of cognitive flexibility and ambiguity tolerance on sense of humor.

Mediating effect pathways	Estimates	95% CI	
		Low	High
Openness to experience→cognitive flexibility→sense of humor	.20*	0.10	0.31
Openness to experience→ambiguity tolerance→sense of humor	0.04	−0.14	0.02
Openness to experience→sense of humor	0.04	−0.13	0.21

*Note*: *p < .05.

### Discussion

Study 1 revealed that an individual's openness to experience does not have a direct impact on their sense of humor, but rather acts as a full mediator through cognitive flexibility. This suggests that increased cognitive flexibility is the underlying mechanism through which openness to experience affects one's sense of humor. However, the ability to produce humor is a variable that highly depends on external evaluation (Martin & Ford, 2018). Whether a person is perceived as humorous ultimately depends on the reactions and recognition of those around them, rather than solely on the speaker's self‐assessment. Therefore, using self‐report methods to measure humor production may not accurately reflect an individual's actual humor production ability.

To further validate the findings of Study 1, Study 2 will utilize humorous dialogue tasks in real‐life situations to measure humor production. After the task is completed, experts will be invited to rate the humor of each participant's responses, serving as an indicator of the participant's humor production ability. The humorous dialogue task has high cognitive complexity and ecological validity and is currently widely used in humor cognition research (Chan et al., 2013; Shibata et al., 2014). This study will provide additional support for the reliability of the results obtained in Study 1.

## STUDY 2

### Methods

#### Participants

A convenient sampling method was used to recruit 218 undergraduate participants in Chengdu of China. The data collection involved a combination of online surveys and paper‐and‐pencil tests. Prior to data collection, informed consent forms in electronic format were obtained from all participants. Thirty‐six participants were excluded from the analysis—due to exhibiting a regular response pattern and a low completion rate on the humor generation test. This resulted in a final sample of 182 valid participants. Among them, 33 (18.1%) were male and 149 (81.9%) were female, with an average age of 19.84 ± 0.75 years. Participants received a cash reward upon completion of the task.

#### Selection and evaluation of humorous dialogue materials

The same measurement tools and methods used in Study 1 to assess openness to experience, cognitive flexibility, and ambiguity tolerance were employed in this study. In terms of the humorous dialogue materials, a total of 103 humorous dialogues were collected. Each dialogue involved two speakers and consisted of two to four sentences. The initial sentences provided the contextual background necessary for generating humor, while the final sentence served as the humorous punchline or “key” that participants were required to generate based on the context. For example:

Person A: “Have you broken off your engagement with Ajuan?”

Person B: “Yeah, she wouldn't marry me!”

Person A: “Did you tell her that your uncle is wealthy?”

Person B: “I did, so now she's my aunt‐in‐law.”

We ensured that the humorous dialogues possessed a regular structure and encompassed diverse content, including sarcastic and aggressive humor. Furthermore, we made sure that the context and answer had open semantic connections to allow participants to generate various humorous responses. Three experts independently rated the humor level and generation difficulty of each dialogue on a 1–7 scale. Based on their ratings, we selected 47 dialogues with a humor score above 5 and a generation difficulty below 3.

To further refine the selection, we recruited 15 participants to engage in real‐time humor generation for the initially screened dialogues. Based on participants' self‐rated scores for the generated key sentences, we ultimately selected 30 humorous dialogues as experimental materials. These dialogues exhibited an average humor rating exceeding 4. These humorous dialogues have been applied in the investigation of the dynamic cognitive processes of humor generation and have demonstrated good reliability and validity (Sun et al., 2024).

#### Procedures

The formal testing in this study took place in a classroom setting. Initially, participants used their mobile phones to complete an online questionnaire assessing openness to experience, cognitive flexibility, and ambiguity tolerance, similar to the procedure in Study 1. Following this, they proceeded to a paper‐and‐pencil test for humor production, consisting of 30 questions. To ensure consistent completion times, participants were instructed to complete the test within a controlled time frame of 20 min. This time constraint was determined based on the evaluation results of our previous humor materials.

#### Processing of data

To evaluate the humor production of the participants, three experts were invited to rate the generated key sentences on a 1–7 scale. The average score from the three experts was used as the humor production score for each participant. The inter‐rater reliability coefficient among the three experts was .817, indicating a high level of consistency in their ratings. Similar to Study 1, gender was used as a covariate in the statistical analysis to account for the gender imbalance in the sample.

### Results

#### Descriptive statistical analysis

Descriptive statistical analysis reveals the means, standard deviations, and inter‐variable correlations, as shown in Table 3. Similar to Study 1, individual openness to experience is significantly positively correlated with ambiguity tolerance, cognitive flexibility, and humor production. Ambiguity tolerance is also significantly and positively correlated with cognitive flexibility and humor production.

**TABLE 3:** Descriptive statistics and correlation analysis between variables (*N* = 182).

	M *±* SD	1	2	3	4
1 Openness to experience	3.49 ± .33	1			
2 Ambiguity tolerance	3.62 ± .72	0.33**	1		
3 Cognitive flexibility	4.01 ± .60	0.43**	0.43**	1	
4 Humor production	3.51 ± .52	0.27**	0.23**	0.06	1

*Note*: **p < .01.

#### The mediating effect of cognitive flexibility and ambiguity tolerance

Similar to Study 1, we also used PROCESS (Model 4) and conducted a parallel mediation analysis using the Bootstrap program with 5000 samples to test for the mediating effects of cognitive flexibility and ambiguity tolerance (Hayes, 2015). Our findings revealed that the mediation effect of openness to experience on humor production through cognitive flexibility had a value of −0.10 with a 95% Bootstrap CI of [−.23, .01], which included “0,” indicating that the mediation effect was not significant. However, openness to experience had a significant mediation effect on humor production through ambiguity tolerance, with a value of 0.11 and a 95% Bootstrap CI of [.02, .22]. Importantly, this interval did not include “0,” suggesting a significant mediation effect. Furthermore, our results indicated that high openness to experience had a direct effect on humor production, with a value of 0.43 and a 95% Bootstrap CI of [.17, .68], which did not include “0,” indicating a significant direct effect. These findings suggest that ambiguity tolerance partially mediates the relationship between high openness to experience and humor production. The relevant results are presented in Table 4.

**TABLE 4:** The mediation analysis on the effects of cognitive flexibility and ambiguity tolerance on humor production.

Mediation effect pathway	Estimates	95% CI	
		Low	High
Openness to experience→cognitive flexibility→humor production	−0.10	−0.23	0.01
Openness to experience→ambiguity tolerance→humor production	.11*	0.02	0.22
Openness to experience→humor production	.43***	0.17	0.68

*Note*: ***p < .001, *p < .05.

### Discussion

In Study 2, we observed that when assessing individuals' humor production ability using an objective task rather than a subjective questionnaire, individuals with higher levels of openness to experience exhibited superior humor production skills. Moreover, we found that the relationship between openness to experience and humor production was partially mediated by tolerance for ambiguity. This implies that individuals with higher levels of openness to experience tend to have greater tolerance for ambiguity, which, in turn, enhances their ability to generate humor.

## GENERAL DISCUSSION

This article examines individuals' potential and ability to produce humor using both subjective and objective methods. Specifically, it explores the influence of the personality trait of openness to experience on humor production and investigates two underlying mechanisms: cognitive flexibility (cognitive path) and ambiguity tolerance (motivational path). Study 1 revealed that openness to experience does not directly enhance sense of humor but operates through cognitive flexibility as a cognitive mechanism. Study 2 demonstrated that individuals with high openness to experience exhibit greater humor production ability compared to those with low openness to experience, with ambiguity tolerance serving as a significant psychological mechanism mediating this relationship.

Individuals with high levels of openness to experience have a positive association with their ability to produce humor. Open individuals possess characteristics that are closely linked to humor generation, including broad emotional experiences, vivid inner fantasies, unique and unconventional values, and a deep appreciation for music, literature, and art (Nusbaum et al., 2014). Their higher verbal vocabulary and capacity to appreciate unconventional elements of humor facilitate the creation and production of humorous language materials. Furthermore, individuals with high openness to experience tend to perceive themselves as more creative and actively engage in creative pursuits (Conner & Silvia, 2015). They also exhibit higher levels of fluid and crystallized intelligence (DeYoung et al., 2014). Interestingly, there is a significant overlap between humor processing and creative cognition, as evidenced by research (Amir et al., 2015; Kozbelt & Nishioka, 2010). Empirical studies have consistently demonstrated a positive correlation between openness to experience and humor generation (Nusbaum, 2015; Nusbaum et al., 2017; Sutu et al., 2021).

Cognitive flexibility and ambiguity tolerance serve as essential psychological mechanisms that contribute to the positive association between openness to experience and sense of humor or humor production ability. Individuals with high openness to experience often demonstrate a preference for novel, varied, and complex stimuli, along with advanced language skills and vocabulary. These motivational and ability‐related characteristics enable them to adapt their perspectives and thinking patterns flexibly when confronted with uncertain situations. Their enhanced cognitive flexibility allows for fresh and innovative approaches to situations and problems, resulting in novel and captivating viewpoints. Research suggests that cognitive flexibility plays a vital role in problem‐solving situations that require insight (Yao et al., 2008). Furthermore, individuals with high openness to experience tend to exhibit greater tolerance for ambiguous situations that involve uncertain information, likely stemming from their inclination toward novel and complex stimuli. The nature of humor production tasks, characterized by ill‐defined problem properties and the non‐uniqueness of humor responses, necessitates a higher level of tolerance for ambiguity.

Our research has revealed distinct psychological mechanisms underlying the relationship between individual openness to experience and humor production when different measurement tools are employed. Specifically, when using self‐report humor questionnaires, cognitive factors, such as cognitive flexibility, emerge as important explanatory mechanisms. On the other hand, when utilizing performance‐based generation tasks, the underlying psychological mechanisms primarily reflect tolerance for ambiguity. We posit that these differences in psychological mechanisms may be attributed to the characteristics of the respective measurement tools for humor production. Köhler and Ruch (1996) argue that subjective self‐report measures of humor production primarily capture the frequency at which individuals generate amusing expressions, which can be influenced by social expectations due to the general desirability of humor. However, other researchers hold different perspectives on this matter. Babad (1974) suggests that an individual's performance on humor production tasks may not necessarily reflect their true level of humor, as these tasks often require individuals to improvise humor, whereas humor in everyday life is often spontaneous. In other words, performance‐based humor production tasks can introduce additional variables related to the experimental situation. Some researchers have emphasized that although performance‐based tests of humor production and self‐report measures of humor production have limited overlap in terms of the content measured, both are effective means of assessing humor production, each focusing on different aspects. Specifically, performance‐based tests primarily concentrate on the behavioral and cognitive dimensions of humor production, emphasizing the highest level of humor production ability, whereas self‐report measures place more emphasis on motivation and interpersonal aspects, highlighting typical behaviors associated with humor production (Ruch & Heintz, 2019).

Overall, this study examined the relationship between openness to experience and humor production, revealing the psychological mechanisms underlying this relationship. On one hand, it verified previous studies on the link between openness to experience and humor production. On the other hand, it provided empirical evidence for theoretical conjectures about why individuals with high levels of openness to experience tend to exhibit greater humor production. When openness to experience is used to predict subjective humor production, cognitive flexibility is the primary psychological mechanism; when predicting objective humor production, ambiguity tolerance is the key internal factor. This finding not only enriches theoretical research but also provides practical guidance that can bring tangible benefits in various fields such as education, workplace applications, and social interactions. For example, educators can incorporate tasks that require quick thinking and adaptive responses into their curriculum and activities to cultivate students' cognitive flexibility, thereby enhancing their sense of humor. In the realm of social interactions, individuals can be trained to remain flexible and tolerant in complex and ambiguous social situations, thereby improving their humor production abilities.

## LIMITATIONS AND FUTURE RESEARCH

The present study utilized a combination of subjective humor questionnaires and objective humor production tasks to examine the influence of individual openness to experience on humor production and explore the underlying psychological mechanisms, thereby enhancing the reliability of the findings. However, the study also has some limitations that should be acknowledged.

Firstly, in Study 1, we only observed the mediating role of cognitive flexibility between openness to experience and humor, while in Study 2, we found that ambiguity tolerance mediated the relationship between openness to experience and humor production. Although these results hold theoretical value, they might be influenced by factors such as the small sample size or the omission of additional variables like verbal intelligence. Future studies should aim to increase the sample size and control for potential confounding variables to establish the robustness and generalizability of the findings.

Secondly, the study focused solely on openness to experience as one of the Big Five personality traits, while relatively neglecting extraversion, which has been a topic of considerable debate regarding its impact on humor production. Some researchers argue that individuals with high openness to experience excel in generating verbal humor, whereas those with high extraversion possess an advantage in expressing and conveying humor (Wilt & Revelle, 2019). Therefore, future research could incorporate measures of humor production in real‐life interactive contexts, in addition to verbal humor tasks, to explore performance differences among individuals with varying levels of extraversion across different humor production tasks.

Lastly, while the study investigated the psychological mechanisms underlying the influence of openness to experience on humor production from various angles, it lacks an examination of how openness to experience operates across different stages of humor production. Recent research has revealed that humor generation involves processes akin to creative problem‐solving, encompassing the generation of humorous ideas and the selection of humorous ideas (Sun et al., 2023). Therefore, future studies could employ neuroelectrophysiological methods to further investigate the dynamic effects of openness to experience on humor production at different stages, shedding light on the underlying neural mechanisms.

## CONCLUSIONS

Individuals with higher levels of openness to experience exhibit greater potential and actual abilities in generating humor. The process of creating funny ideas is influenced by two psychological pathways: cognitive flexibility (cognitive path) and ambiguity tolerance (motivational path). The specific pathway through which openness to experience influences humor production depends on the measurement method used. When subjective humor questionnaires are employed, openness to experience primarily impacts the generation of humorous ideas through the cognitive path. Conversely, when objective humor dialogue production tasks are used, openness to experience predominantly affects the generation of humorous ideas through the motivational path.

## CONFLICT OF INTEREST STATEMENT

There is no competing interests with other people or organizations.

## ETHICS STATEMENT

The study was conducted in accordance with the ethical standards outlined in the 1964 Declaration of Helsinki and its subsequent amendments, or equivalent ethical standards. All procedures carried out in the study were approved by the Ethics Committee of Sichuan Normal University.

## INFORMED CONSENT STATEMENT

All study participants provided informed consent.

## Data Availability

The data that support the findings of this study are available from the corresponding author or the first author upon reasonable request. The data are not publicly available due to privacy or ethical restrictions.
